# The Seed Development Factors *TT2* and *MYB5* Regulate Heat Stress Response in *Arabidopsis*

**DOI:** 10.3390/genes12050746

**Published:** 2021-05-15

**Authors:** Pierre Jacob, Gwilherm Brisou, Marion Dalmais, Johanne Thévenin, Froukje van der Wal, David Latrasse, Ravi Suresh Devani, Moussa Benhamed, Bertrand Dubreucq, Adnane Boualem, Loic Lepiniec, Richard G. H. Immink, Heribert Hirt, Abdelhafid Bendahmane

**Affiliations:** 1 Institute of Plant Sciences Paris-Saclay, Université Paris-Saclay, Univ. Evry, INRAE, CNRS, 91405 Orsay, France; jpierre@email.unc.edu (P.J.); gwilherm.brisou@universite-paris-saclay.fr (G.B.); marion.dalmais@inrae.fr (M.D.); david.latrasse@ips2.universite-paris-saclay.fr (D.L.); ravi-sureshbhai.devani@universite-paris-saclay.fr (R.S.D.); moussa.benhamed@ips2.universite-paris-saclay.fr (M.B.); adnane.boualem@inrae.fr (A.B.); 2 Institut Jean-Pierre Bourgin, INRAE, AgroParisTech, Université Paris-Saclay, 78000 Versailles, France; Johanne.Thevenin@inrae.fr (J.T.); bertrand.dubreucq@inrae.fr (B.D.); loic.lepiniec@inrae.fr (L.L.); 3 Bioscience and Laboratory of Molecular Biology, Wageningen University and Research, 6708PB Wageningen, The Netherlands; froukje.vanderwal@wur.nl (F.v.d.W.); Richard.Immink@wur.nl (R.G.H.I.); 4 Darwin21, Biological and Environmental Science and Engineering Division (BESE), King Abdullah University of Science and Technology, Thuwal 23955-6900, Saudi Arabia; heribert.hirt@kaust.edu.sa; 5 Max Perutz Laboratories, University of Vienna, 1030 Vienna, Austria

**Keywords:** environmental stress, seed development, site II element, HSFA2, TT2/ MYB5-MBW complex

## Abstract

*HEAT SHOCK FACTOR A2* (*HSFA2*) is a regulator of multiple environmental stress responses required for stress acclimation. We analyzed *HSFA2* co-regulated genes and identified 43 genes strongly co-regulated with *HSFA2* during multiple stresses. Motif enrichment analysis revealed an over-representation of the site II element (SIIE) in the promoters of these genes. In a yeast 1-hybrid screen with the SIIE, we identified the closely related R2R3-MYB transcription factors TT2 and MYB5. We found overexpression of *MYB5* or *TT2* rendered plants heat stress tolerant. In contrast, *tt2*, *myb5*, and *tt2/myb5* loss of function mutants showed heat stress hypersensitivity. Transient expression assays confirmed that MYB5 and TT2 can regulate the *HSFA2* promoter together with the other members of the MBW complex, TT8 and TRANSPARENT TESTA GLABRA 1 (TTG1) and that the SIIE was involved in this regulation. Transcriptomic analysis revealed that *TT2/MYB5* target promoters were enriched in SIIE. Overall, we report a new function of TT2 and MYB5 in stress resistance and a role in SIIE-mediated *HSFA2* regulation.

## 1. Introduction

Heat shock factors (HSFs) represent a widely conserved class of transcription factors involved in stress response and development [1,2]. Although they were first discovered in the context of the heat shock response, most biotic and abiotic stress responses require the concerted action of HSFs to regulate stress response and acclimation [3,4]. Elucidating the molecular mechanisms responsible for the regulation of *HSFs* is critical to enhance stress tolerance of plants. 

Plant genomes contain a large number of *HSFs*; 21 in *Arabidopsis*, 25 in rice or 38 in soybean, compared to a single *HSF1* in *Saccharomyces cerevisiae* and seven in humans [5]. HSFs are grouped into three classes depending on the presence of specific protein domains/motifs. A class HSFs positively regulate gene expression as they exhibit the transcription activator motif AHA (aromatic and large hydrophobic residues in an acidic context), whereas B and C class HSFs are considered to function as transcriptional inhibitors or co-activators.

In *Arabidopsis thaliana*, *HSFA2* seems to be of particular importance [3]. Whereas the master regulators of the HSF pathway, the 4 *HSFA1s* are specifically modulated by different environmental cues, *HSFA2* is induced systemically. *HSFA2* overexpression (OE) is sufficient to rescue most of the *hsfa1s* quadruple mutant defects [6,7]. Accordingly, *HSFA2* OE leads to resistance against multiple environmental stresses [8,9,10]. 

Among *Arabidopsis* A-class *HSFs*, HSFA1d and HSFA1e have been found to regulate *HSFA2.* However, *HSFA2* is still highly induced in response to heat and/or high light stress in the double *hsfa1d/hsfa1e* mutant [11]. Consequently, at least one other positive regulator of *HSFA2* expression must exist. 

In this study, we compared genes co-regulated with *HSFA2* under different stress conditions to define a cluster of *HSFA2* coregulated genes. We identified 43 genes strongly coregulated with *AtHSFA2* during cold, salt, heat, and hypoxia stress. Promoter analysis revealed the site II element (SIIE) to be enriched in the promoters of these genes. A yeast one hybrid (Y1H) screen, using *HSFA2* promoter as a bait, led to the identification of two close paralogs, the R2R3-MYB transcription factors TRANSPARENT TESTA 2 (TT2) and MYB5 as putative SIIE-binding proteins (SIIEBP). 

*TT2* was identified as a seed coat-specific factor responsible for proanthocyanidin (PA) accumulation, giving *Arabidopsis* seeds their characteristic brown color [12]. The function of TT2 in PA accumulation requires its interaction with a bHLH (TT8) and a WDR protein (TRANSPARENT TESTA GLABRA1 [TTG1]) to form an MBW (MYB/bHLH/WDR) complex that regulates several late anthocyanin/PA biosynthetic genes (LBGs). Multiple MBW complexes exist, with TT2 or the closely related MYB5 protein that differentially regulate LBGs including *BANYULS* (*BAN*) [12,13,14,15,16,17]. Even though MYB5 and TT2 are acting in similar protein complexes, they are spatially separated and not functionally redundant. *Myb5-1* mutants do not exhibit the transparent testa phenotype and most notably are defective in seed coat mucilage [18]. In MBW complexes, TT2 and MYB5 have been shown to interact with MYB core [C/T]NGTTR and/or and AC-rich elements, [A/C]CC[A/T]A[A/C], whereas bHLH partners bind E-box motifs (CANNTG/CACGTG) [17]. 

While *TT2* was previously described as a seed-specific factor, we found *TT2* along with its targets is induced in vegetative organs by heat stress. We show that TT2/MYB5-mediated *HFSA2* regulation involves the SIIE cis-element, in yeast and in planta. We also showed that TT2/MYB5-mediated *HFSA2* expression is significantly enhanced in the presence of TT8 and TTG1, two other members of the MBW complex. Consistent with this result, *Arabidopsis* plants overexpressing *TT2* and, to a lesser extent *MYB5*, exhibit enhanced resistance to heat stress and *tt2*, *myb5, tt8* and *tt2/myb5* loss of function mutants are more sensitive to heat stress. Gene expression analyses further confirm that *TT2* and *MYB5* upregulate genes related to multiple stress responses. Promoter enrichment analysis of differentially regulated gene networks revealed two different modes of transcriptional regulation, one depending on the AC-rich element and the E-box (secondary metabolic process related genes), and the other involving the SIIE, the E-box, and the HSE (genes involved in stress response). Overall, we report a new function of *TT2* and *MYB5* in stress response. 

## 2. Materials and Methods

### 2.1. Plant Material

Experiments were performed on *Arabidopsis thaliana* accession *Columbia 0*, unless specified otherwise. Plants were grown in growth chamber with a 100 µmol.m^−2^.s^−1^ light intensity with 8 h to 16 h of illumination per day for short and long day photoperiods, respectively. T-DNA insertion mutants *tt8-6* (N2105594), *tt2-5* (N2105593), *tt2-1* (NW83), and *myb5-1* (N2106725) were obtained from the NASC. The *tt2/myb5* double mutant was obtained from a cross between *tt2-1* and *myb5-1* and a homozygote individual was backcrossed three times in Col 0. Sequence data from this article can be found in The Arabidopsis Information Resource under the following accession numbers: AT2G26150 (*HSFA2*), AT3G35550 (*TT2*), AT3G13540 (*MYB5*), AT5G24520 (*TTG1*), AT4G09820 (*TT8*), AT1G61720 (*BAN*), AT4G22880 (*LDOX*), AT5G42800 (*DFR*), AT1G56650 (*PAP1*).

### 2.2. Bio-Informatic Analyses

Genevestigator software was used for publicly available microarray data analyses [19]. Coregulated genes were determined using the coregulations tool with a 0.8 Pearson’s coregulation coefficient cutoff. Genes’ descriptions and promoter sequence consensus were the ones of TAIR10 (www.tair.com, accessed on February 2016). Promoter motif enrichment was analyzed using the MEME suite V4.12.0 [20]. MEME was used for de novo motif prediction and AME was used for detecting the enrichment of known motif. 

### 2.3. Yeast One Hybrid

The REGIA collection was used for the screening, as previously described [21]. Site II element was de novo synthesized with cohesive ends corresponding to HindIII recognition site (Eurofins, Hamburg, Germany). Vector was digested accordingly and the following sequences were inserted in the bait vector by ligation: forward 5’ agc ttT CGT TAG AAA TAT ATT TAA GTA AAG TAT ATT ATG ATA TAT Ac 3’ and reverse 5’ tcg agT ATA TAT CAT AAT ATA CTT TAC TTA AAT ATA TTT CTA ACG Aa 3’. Cohesive ends are in lower case characters. The identity of the prey was confirmed by PCR followed by SANGER sequencing using target specific primers (Supplementary Table S1). 

### 2.4. Plant Transformation 

Coding sequences were amplified using the primers in Supplementary Table S10 (lower case indicates attB recombination sites). Amplified fragments were cloned into pDNR207 using the Gateway BP Clonase II enzyme mix (Thermo Fisher scientific n°11789020, Waltham, MA, USA) and then into a modified pGREENII0229 containing a gateway cassette using the Gateway LR Clonase II Enzyme mix (Thermo Fisher Scientific n°11791100, Waltham, MA, USA). Vectors were then transformed into *Agrobacterium tumefaciens* pMP90. Plant transformations were performed following the floral dip method.

### 2.5. Transient Protoplast Transformation and Flow Cytometry

*TT2* and *MYB5* coding sequences were amplified and cloned in pDNR207 entry vector with the BP clonase II mix (Thermo Fisher Scientific n°11789020). They were subsequently cloned in pBluescript-derived, *Physcomitrella patens* expression vectors [22], using the Gateway LR clonase II enzyme mix (Thermo Fisher Scientific n°11791100). The *HSFA2* promoter was amplified from Col 0 genomic DNA and cloned in pDNR207 entry vector with the BP clonase II mix (Thermo Fisher Scientific n°11789020). The promoter was then introduced into the destination GFP expression vector with the Gateway LR clonase II enzyme mix (Thermo Fisher Scientific n°11791100). The mutated HSFA2 promoter was synthesized by Eurofins genomics. WT promoter containing vector and the mutated promoter were digested by AscI and EcoRV and the mutated promoter was introduced in the GFP expression vector by ligation. Protoplast were prepared as previously described [22], and transformed with 5 µg of each intended plasmid. Protoplast fluorescence was determined 48 h after transformation using flow cytometry. Protoplast suspensions were filtered through a 30-μm mesh. Flow cytometry was performed on a Partec CyFlow® Space instrument (Sysmex France, 93420 Villepinte, France), with a 488 nm solid sapphire 20 mW laser for excitation and using a FloMax® data acquisition and analysis software (Sysmex France, 93420 Villepinte, France). Green fluorescence was detected with a FITC 527nm/30nm band-pass filter (FL1 channel). Red chlorophyll-based fluorescence from living protoplasts was detected with a 610nm/30nm band-pass filter in the FL2 channel. The side light scatter (SSC) detector high voltage was set to 161.5 V. The photomultiplier tube voltages were adjusted to 275 V for FL1 and 475 V for FL2 (logarithmic amplification mode, four decades range, speed 4). For each sample, the GFP fluorescence per population of cells was calculated as the product of the average fluorescence intensity by the number of cells in the positive gate, normalized by the total number of living protoplasts in the transformation. The gate was drawn along a line of maximum GFP intensities for positive samples, when compared with protoplasts that were only transfected with *pBAN*:*GFP* as negative controls. 

### 2.6. Heat Stress Resistance Assays

Seeds were gas-phased sterilized from 4 to 8 h. They were placed in an open tube inside a hermetic box containing a beaker with 50 mL of 9.6% bleach topped with a basket containing 3 mL of 37% HCl (Sigma-Aldrich, 258148, St. Louis, MO, USA). The box was sealed and shacked, and the HCl was poured into the bleach. After 4 to 8 h of sterilization, the seeds were sowed in 90 mm petri dishes on half strength Murashige and Skoog (MS) basal salt mixture (Sigma-Aldrich, M5524, St. Louis, MO, USA). The seeds were stratified at 4 °C for 48 h and seedlings were grown for 6 days in short day conditions before applying stress. Heat challenge consisted of 80 min at 44 °C. Plants were put back to control conditions immediately after stress and allowed to recover in control conditions for 10 days before survival rate was determined. Plants were considered dead if completely bleached, collapsed, or presenting a translucent, necrotic aspect.

### 2.7. RNA Extraction and Q-PCR

In vitro grown plantlets were used in all Q-PCR experiments. Plants were gathered and RNA was extracted with the Qiagen RNeasy plant mini kit (Qiagen, cat 74903, Hilden, Germany). The “user-developed protocol” for plant tissue was used without modifications. 1 µg of total RNAs were reverse transcribed using the Superscript II kit (Invitrogen, 18064, Carlsbad, CA, USA), according to the manufacturer’s instructions. mRNAs were quantified by Q-PCR using the MESA GREEN qPCR MasterMix (Eurogentec, RT-SY2X-03+WOU, Liege, Belgium). Actin and 26S proteasome mRNA were both used as reference genes in all experiments. Runs were performed on the CFX384 Touch™ Real-Time PCR Detection System and relative mRNA levels were analysed using the software Bio-Rad CFX manager (http://www.bio-rad.com, accessed on February 2016). Primers used for Q-PCR are presented in Supplementary Table S2.

### 2.8. RNA-Sequencing Transcriptomic Analysis 

Libraries were built from 2 µg of total RNA with the NEBNext® Ultra™ Directional RNA Library Prep Kit for Illumina (ref#E7420S) and sequenced on the Illumina NextSeq 500. Reads mapping and statistical analysis was performed with CLC Genomics Workbench 10 RNA-seq analysis suite. Gene ontology analyses were performed with AgriGO (http://bioinfo.cau.edu.cn/agriGO, accessed on August 2017). Gene regulatory network were modeled with Genemania (https://genemania.org/, accessed on August 2017). De novo motif discovery and motif enrichment analyses were performed with the MEME suite V4.12.0.

## 3. Results

### 3.1. Promoters of HSFA2 Coregulated Genes Are Enriched in the Site II Element Motif

**Figure 1:**
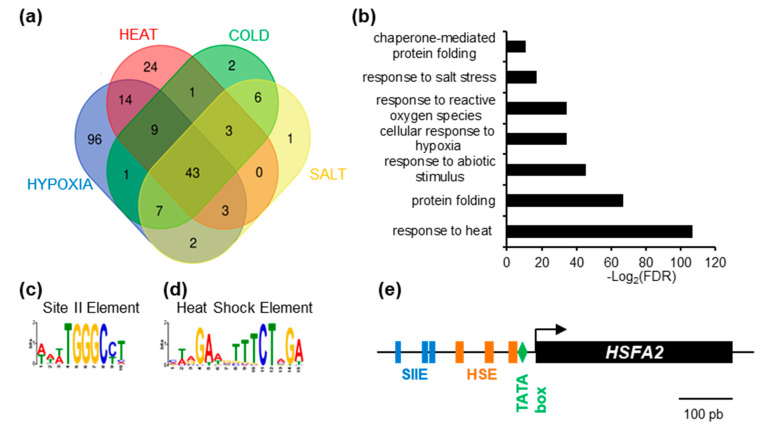
Identification of enriched cis-element in the promoter of HEAT SHOCK FACTOR A2 (HSFA2)-coregulated genes. (**a**) Venn diagram representing the intersection between genes identified as positively regulated with HSFA2 during cold, salt, heat, and hypoxia stress. (**b**) GO term enrichment of the 43 genes in the HSFA2 common stress cluster. (**c**) SII element (WWWTGGGCCT) and (**d**) Heat Shock Element (MWRGAWGTTTCTAGA) DNA motifs enriched in the promoter of the 43 *HSFA2* common stress cluster. (**e**) Schematic representation of *HSFA2* promoter featuring TATA box (−23), HSEs (−33; −77; −133) and SII (−186; −201; −250).

To gain insight into the regulation of *HSFA2*, we used publicly available gene expression data (www.genevestigator.com, accessed on October 2015 [19]) and searched for *HSFA2* co-regulated genes. Genes showing a Pearson correlation coefficient above 0.8 were extracted. As expected, *HSFA2* was part of expression clusters strongly correlated during heat treatment (101 genes), cold stress (75 genes), hypoxia (178 genes), and salt stress (67 genes). One of the clusters, consisting of 43 genes, is systematically co-regulated with *HSFA2* and therefore called “*HSFA2* common stress cluster” (Figure 1a, Supplementary Table S3). The *HSFA2* common stress cluster is enriched in gene ontology terms “response to stress” and “protein folding” (Panther^TM^ V12) and comprises several known HSFA2 targets (Figure 1b, Supplementary Table S1). 

Search for cis-elements in the promoters of the *HSFA2* common stress cluster, using the MEME suite software [20] revealed two significantly over-represented motifs, the Heat Shock Element and the Site II element, hereafter called HSE and SIIE, respectively (Figure 1c,d). The HSE (5’-nGAAn-3’ repeats) was previously shown to be enriched in the promoters of HSFA2 and its targets [7,23], thereby validating the method (Figure 1d). Unexpectedly, we found that the majority (65.91%) of promoters (*p* = 4.4 × 10^−12^, Figure 1c) were also enriched in sequences corresponding to the SIIE motif (5’-(A/T)TGGGC(C/T) -3’ [24]. Analysis with PLMdetect further pinpointed an enrichment of the SIIE motif in a 200 bp window, between −136 bp and −332 bp upstream of the TSS [25]. Consistent with motifs involvement in transcription regulation, SIIEs were often found several times in the same promoters, in both forward and reverse orientation (Supplementary Figure S1). In the *HSFA2* promoter, 3 SIIEs were found in close proximity to the TSS (Figure 1e).

The SIIE was previously linked to cell cycle regulation as well as biotic and abiotic stress responses [24,26]. However, the identity of SIIE binding factors is still under debate. It was suggested that SIIEs interact with the TEOSINTE BRANCHED-1/CYCLOIDEA/PCF 20 (TCP20) factor [27]. However, in vivo experiments showed that TCP20 was not present in the region of SIIE motifs [28]. 

### 3.2. TT2 and MYB5, Two Related R2R3-MYBs TFs, Bind to HSFA2 Site II Element in Yeast

**Figure 2:**
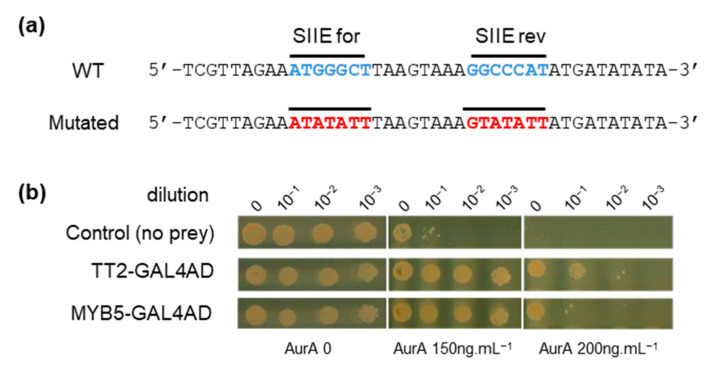
**TRANSPARENT TESTA 2** (TT2) and MYB5 bind to the HEAT SHOCK FACTOR A2 (HSFA2) promoter. (**a**) Wild type (WT) and mutated sequences of the HSFA2 promoter used for the screening. (**b**) Yeast 1 hybrid interaction strength of TT2 and MYB5 with the HSFA2 promoter at different aureobasidin A, AurA, concentrations.

To identify upstream transcriptional regulators that could drive HSFA2 expression through binding of SIIE, we carried out a yeast one-hybrid (Y1H) screen. A 40 bp region of the *HSFA2* promoter from 175 to 216 bp before the TSS and containing two SIIEs, in sense and antisense orientation, was used as bait (Figure 2a). A similar sequence mutated on the SIIEs was used as a negative control to ensure the specificity of the interaction (Figure 2a). The REGIA collection, containing 1357 *Arabidopsis* transcription factors cloned in *Saccharomyces cerevisiae*, provided the set of different preys in the Y1H screen [21]. Clones harboring the mutated bait were subtracted from the list of positive clones possessing the wild type (WT) bait. Two TFs, TT2 (TRANSPARENT TESTA 2) and MYB5, fused to the GAL4 activation domain (GAL4AD), were identified to specifically activate transcription through SIIEs. To get an estimate of the strength of the interaction, yeast suspensions were diluted 10, 100, and 1000 fold and spotted on increasing concentrations of the antibiotic aureobasidin A (AurA), the reporter used for the screen. Growth inhibition was observed for the negative control (yeast with the bait but not the prey) at the standard concentration of 150 ng.µL^−1^, and was complete at 200 ng.µL^−1^ AurA (Figure 2b). Yeast harboring SIIE as bait and the prey TT2-GAL4AD or MYB5-GAL4AD showed no growth inhibition at 200 ng.µL^−1^ AurA. Dilution experiments in the yeast cellular context further indicated that TT2 has a higher affinity for SIIEs than MYB5 (Figure 2b). Interestingly, TT2 and MYB5 are closely related transcription factors belonging to R2R3-MYBs. 

### 3.3. TT2 or MYB5 Overexpression Induces HSFA2 Expression and Provides Stress Resistance in Planta 

**Figure 3:**
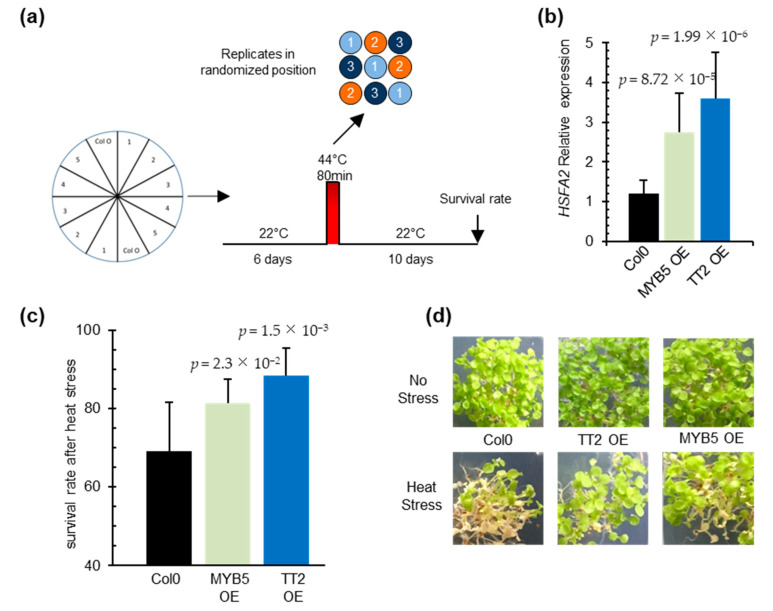
**TRANSPARENT TESTA 2** (TT2) and MYB5 overexpression triggers upregulation of HEAT SHOCK FACTOR A2 (HSFA2) in planta and provides heat resistance. (**a**) Schematic representation of the resistance assay protocol. Each stress is applied on three identical petri dishes placed in randomized positions in the growth chamber. Lines were sowed symmetrically in vitro and grown in control conditions for six days. Heat stress consisted of 80 min at 44 °C. Survival rates were observed after 10 days. (**b**) HSFA2 expression in transgenic *TT2* or *MYB5* overexpressors (OEs) plants. Results are the mean of three independent measurements. Errors bars represent standard deviations (SD). Results are significant with *p*-values < 0.05 (Wilcoxon test). (**c**) Survival rates of TT2 OEs and MYB5 OEs plants after heat stress. Histograms present the mean of three independent measurements. Each measurement is the average of six randomized technical replicates. Error bars represent SD. *p*-values were obtained from Student’s *t*-test. (**d**) Representative pictures of two-week-old TT2 OEs and MYB5 OEs plants grown in control and heat stress conditions.

To investigate the function of *TT2* and *MYB5* in heat stress, we constitutively expressed the two TFs under the control of CaMV35S promoter in *Arabidopsis*. Three and four independent transgenic lines, exhibiting strong *TT2* and *MYB5* transgene expression, respectively, were recovered (Supplementary Figure S2a,b). Analysis of *HSFA2* transcript accumulation in six-day-old seedlings revealed the constitutive expression of *HSFA2* in both *TT2* or *MYB5* overexpressor lines (Figure 3b). In accordance with previous reports describing *MYB5* as a ‘weak’ transcriptional activator [29], 35S:*TT2* plants accumulated more *HSFA2* transcripts than 35S:*MYB5* plants in control conditions (Figure 3b). As constitutive expression of *HSFA2* leads to thermotolerance, we assessed 35S:*TT2* and 35S:*MYB5* lines for heat stress resistance. Heat stress was applied on six-day-old seedlings and consisted of incubation of the seedlings for 80 min at 44 °C. To minimize experimental variability, plants were sown twice per plate, symmetrically, and in three randomized technical replicates (Figure 3a). Both 35S:*TT2* and 35S:*MYB5* lines showed enhanced resistance to heat stress in three independent biological replicates (Figure 3c,d, Supplementary Figure S2d). The heat stress resistance phenotype was stronger for 35S:*TT2* than for 35S:*MYB5* lines. Interestingly, no obvious developmental defects were observed in neither *TT2* nor *MYB5* overexpression plants in control conditions (Figure 3d, Supplementary Figure S2c). From this, we concluded that *TT2* and *MYB5* regulate *HSFA2* expression and lead to heat stress resistance.

### 3.4. TT2, MYB5, TT8, and TTG1 Activate HSFA2 Expression via SIIE Motifs

It has been shown that *TT2* and *MYB5* control outer seed coat development via a ternary MYB–BHLH–WDR (MBW) protein complex involving TT2⁄AtMYB123 or MYB5, TT8⁄AtBHLH042 and TTG1(TRANSPARENT TESTA GLABRA 1: WD-repeat protein) [29,30]. To test whether *TT2* and *MYB5* mediated *HSFA2* expression via SIIE involves the MBW complex, we exploited the *Physcomitrella patens* protoplast transient expression system. This consists of co-transformation of moss protoplasts with the investigated promoter and the test transcription factors. For quantification of the strength of the interaction, this method combines the advantages of GFP as a marker of promoter activity as a fast and reliable method for fluorescence measurements in cells with flow cytometry [22].

**Figure 4:**
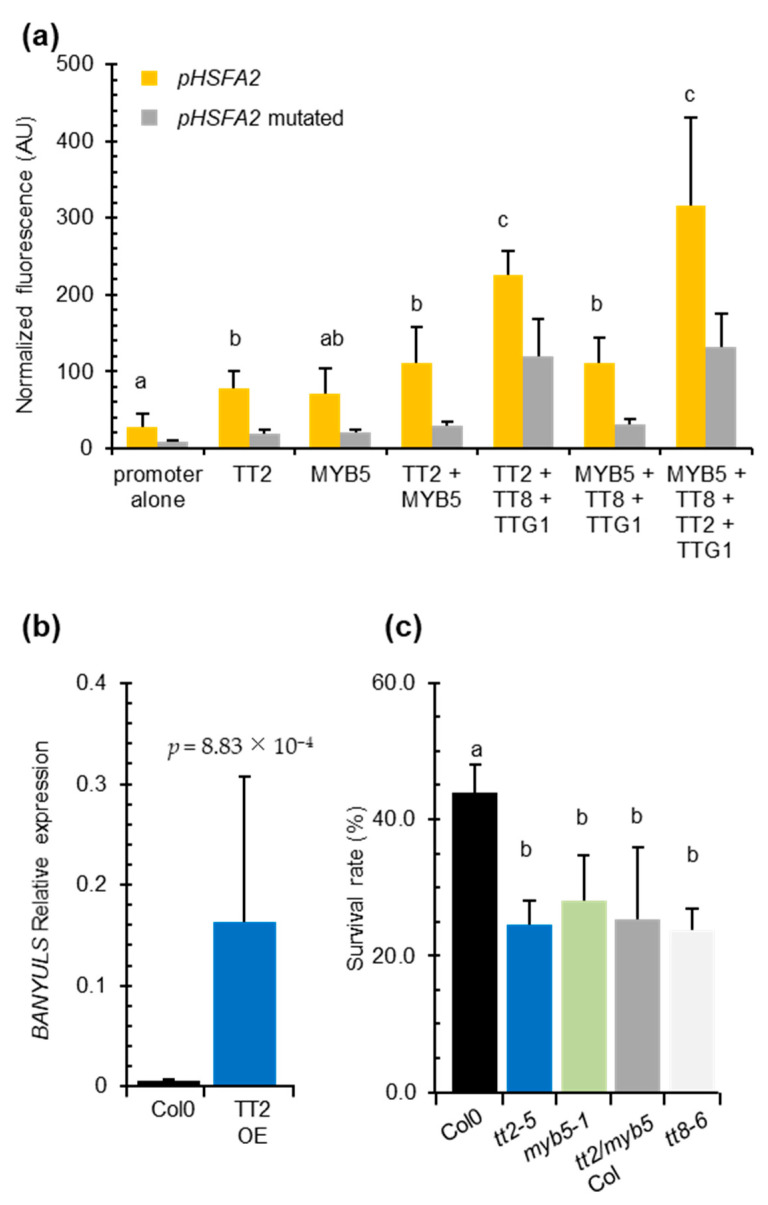
The entire MYB–BHLH–WDR (MBW) complex is required for *HEAT SHOCK FACTOR A2* (*HSFA2*) regulation and heat stress resistance in vegetative organs. (**a**) *Physcomitrella patens* protoplast transient transformation assays. *HSFA2* promoter or the mutated *HSFA2* promoter version lacking the three SIIelement were co-transformed in moss protoplasts with different transcription factors composing the MBW complex. Total fluorescence was normalized by the number of protoplasts per transformation. Differences between wild type and mutated promoter activities are all significantly different with *p*-values < 0.03. (**b**) *BANYULS* (*BAN*) expression in five independent *TRANSPARENT TESTA 2* (*TT2*) overexpressors. (**c**) Heat stress resistance potential of Col-0 compared to *tt2-5* and *myb5-1* and the double mutant *tt2/myb5* backcrossed three times in Col-0 (*tt2/myb5 Col).* Histograms are the mean of three independent experiments, each consisting of the average of six randomized technical replicates. *p*-values were obtained by the Student’s *t*-test and error bars represent SDs. Letters indicate statistical significance with *p*-values < 0.05.

We co-transformed *P. patens* protoplasts with the *HSFA2* promoter GFP reporter construct p*HSFA2*:*GFP* in combinations with vectors expressing *TT2, MYB5*, *TT8*, or *TTG1*. As control, we used the *pHSFA2mut*:*GFP* reporter construct mutated in the SIIEs. The *HSFA2* promoter alone exhibited significant autoactivity that was reduced three-fold when the SIIEs were mutated (Figure 4a). This result indicated that homologous moss transcription factors are able to bind the SIIEs. Co-expression of *TT2* with *pHSFA2:GFP* increased the GFP signal 2.5-fold (Figure 4a). A similar increase was observed when *MYB5* was co-expressed with *pHSFA2:GFP* (Figure 4a). Both signals were significantly decreased when *TT2* or *MYB5* were co-expressed with the mutated promoter construct *pHSFA2mut:GFP* (Figure 4a). These results indicate that the SIIEs are necessary for the correct regulation of the *HSFA2* promoter by *TT2* or *MYB5*. Co-expression of *TT2* and *MYB5* further increased the activity of the *HSFA2* promoter, leading to a 3.9 fold increase of the GFP signal (Figure 4a). Consistent with previous reports describing MYB5-driven transcription as relatively weak [29], *MYB5/TT8/TTG1* co-expression triggered a 3.9-fold increase in GFP, whereas *TT2*/*TT8*/*TTG1* induced an 8-fold increase. The strongest signal was obtained by the *TT2*/*MYB5*/*TT8*/*TTG1* combination that yielded a 10-fold increase in GFP signal (Figure 4a). These results further confirmed that TT2, TT8, and TTG1 and to a lesser extent MYB5, cooperatively regulate the *HSFA2* promoter. Furthermore, GFP signals were weak when the different combinations of transcription factors were co-transformed with the promoter that lacks the SIIEs, indicating that the MBW complex activates *HSFA2* expression via SIIEs (Figure 4a).

### 3.5. The TT2/MYB5-MBW Complex Is Active in Vegetative Organs and Is Required for Heat Stress Resistance

We found that the TT2/MYB5-TT8-TTG1 complex can modulate the activity of the *HSFA2* promoter in transient assays and in transgenic overexpression lines (Supplementary Figures S3 and S4a). However, this complex was previously described as seed coat-specific [17]. To check whether the MBW complex could be active outside the seed context and responsible for the heat stress resistance phenotype, we looked for *BAN* transcript accumulation, a target of the MBW complex, in *TT2* OE lines by Q-PCR. We reverse transcribed RNA extracted from six-day-old in vitro grown plants. Consistent with previous reports describing the effect of *TT2* ectopic expression in roots and seedlings [12], we found *TT2*-overexpressing lines constitutively accumulate *BAN* transcripts in the absence of stress (Figure 4b). This confirmed that all the requirements for TT2-containing MBW complex activity were also met in vegetative organs. 

To test if TT2/MYB5-mediated *HSFA2* expression has a biological role in heat stress resistance and is not an artifact of overexpression, we subjected *tt2-5*, *myb5-1* and *tt2-1/myb5* loss of function mutants to heat stress. As *tt2-1* and *myb5* derive from *Ler* and Col-0 ecotypes, respectively, the double mutant was backcrossed three times in Col-0 to generate *tt2/myb5* double mutants in a genetic background approaching *Col*-0. Both the simple, *tt2-5*, *myb5-1* and the double *tt2-1/myb5* mutants were compromised in basal heat stress resistance when compared to the wild type control Col-0 (Figure 4c). The heat stress sensitivity was also observed for the *tt2-1* mutant in the *Ler* background (Supplementary Figure S2e). 

To investigate if *TT2* and *MYB5* function in heat resistance required the full MBW complex, we phenotyped *TT8* loss of function mutant, *tt8-6*, for heat resistance. TT8 is a central protein in the MBW ternary protein complex that is expressed in both seeds and vegetative tissues. Consistent with the *HSFA2* promoter-MBW interaction in *P. patens* protoplast transient expression assays, we found that basal heat stress resistance was compromised in *tt8-6* mutant plants (Figure 4c). As both *tt2/myb5* and *tt8* loss of function mutants are compromised in basal heat stress resistance, we concluded that the MBW ternary complex likely controls expression of genes required for basal heat stress resistance in *Arabidopsis*. 

**Figure 5:**
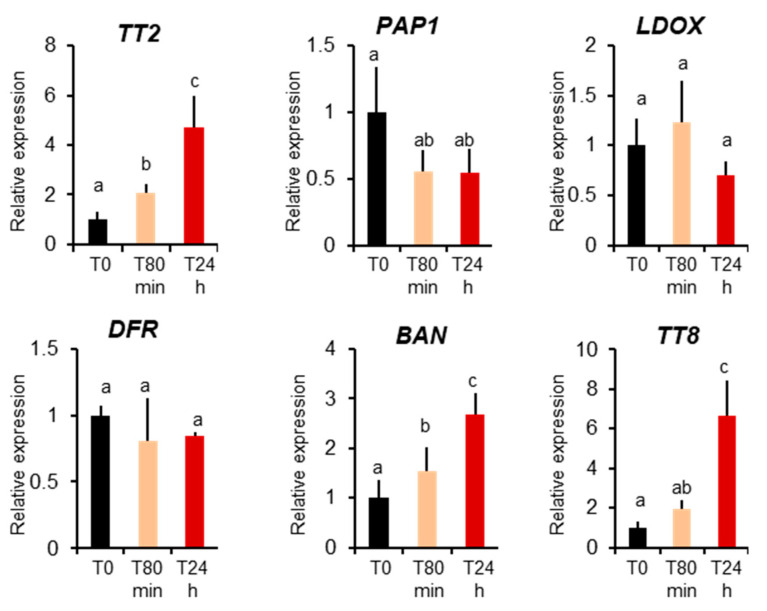
MYB–BHLH–WDR component and target expression during heat stress. Col 0-plants were collected before heat stress (T0), after 80 minutes of heat stress (T80min) and the next day (T24h). Gene expression was measured using real-time quantitative PCR. Results are the means of six independent experiments. Bars represent standard errors. Different letters indicate the statistical significance with *p*-values < 0.05 (Student’s *t*-test).

Previously only MBW complexes containing Production of Anthocyanin Pigments 1 protein, PAP1, were described to be active in vegetative organs [17]. To further confirm that the TT2-containing MBW complex is active in leaves during heat stress, we investigated *TT2* and *PAP1* expression before and 80 minutes and 24 hours after heat stress treatments. We detected a very low level expression of *TT2* in plants before stress, but a four-fold upregulation upon heat stress. Inversely, we observed downregulation of *PAP1* during heat stress (Figure 5). To quantify the activity of TT2 and PAP1 during stress, we analyzed the expression of their direct targets, using Q-PCR. PAP1 induces the expressions of *LDOX*, *DFR*, and *TT8* but not *BAN*, whereas the expression of *LDOX*, *DFR*, *BAN*, and *TT8* is induced by TT2 [17]. Only *BAN* and *TT8* transcripts showed a marked increase after heat stress. On the contrary, the non-specific *DFR* and *LDOX* were not found modulated by heat stress (Figure 5). Taken together, these results show the TT2/MYB5-MBW complex is active in vegetative organs under heat stress.

### 3.6. TT2 and MYB5 Redundantly Regulate Stress Response Genes

**Figure 6:**
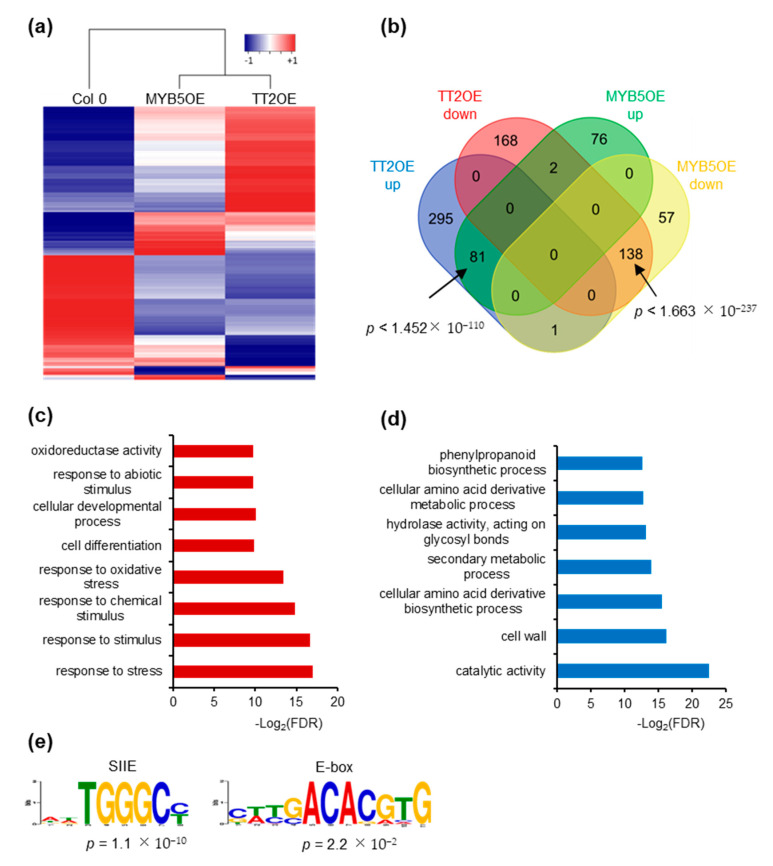
TRANSPARENT TESTA 2 (TT2) overexpressor (OE) and MYB5 OE redundantly regulate stress response genes. (**a**) Heatmap representation of *TT2* OE and *MYB5* OE up- (red) and downregulated (blue) genes. Heatmap was generated from mean RPKM values. (**b**) Venn diagram showing the overlap between *TT2* OE and *MYB5* OE differentially expressed genes, DEGs. 51.3% of the *MYB5* OE upregulated genes are also upregulated in *TT2* OE and 70.4% of the *MYB5* OE downregulated genes are also downregulated in *TT2* OE. The overlaps are statistically significant, hypergeometric test. (**c**) and (**d**) Gene ontology (GO) term enrichment analysis of common upregulated (**c**) or downregulated (**d**) genes in *TT2* and *MYB5* OEs. (**e**) DNA motif enriched in the promoter of genes upregulated in TT2 OEs plants.

We further investigated the impact of *TT2* and *MYB5* overexpression on gene expression through RNA-sequencing. We subjected unstressed, six-day-old Col-0, *TT2* OE and *MYB5* OE plants from three independent biological replicates to total RNA extraction and sequencing. We obtained approximately 40 million paired-end reads from each library. Reads were mapped and used to estimate gene expression using the RNAseq analysis suite of CLC genomics workbench (Qiagen). Compared to Col-0, *TT2* OE and *MYB5* OE showed 684 and 354 differentially expressed genes (DEGs), respectively, with FDR adjusted *p*-values under 0.05 (Supplementary Table S4). The overexpressed *TT2* or *MYB5* transcripts were excluded from subsequent analyses. Most DEGs (62.7%), in *MYB5* OE were similarly regulated in *TT2* OE (Figure 6a,b). Eighty-one genes were upregulated in both *TT2* OE and *MYB5* OE. Gene ontology (GO) analysis of those upregulated genes indicated enrichment in the “response to stress” or related categories (response to stimulus, abiotic stress, oxidative stress) as well as the “cellular development” and “cell differentiation” categories (Figure 6c). Interestingly, among the flavonoid biosynthesis genes, *LDOX, TTG2* were upregulated and *FLAVONOL SYNTHASE 1* (*FLS1*) was downregulated in *TT2* and *MYB5* OE. *TT4*, *TT8*, *BAN*, and *DFR* were found upregulated in *TT2* OE only. 

Downregulated genes in *TT2* OE and *MYB5* OE were enriched in “secondary metabolic process”, “phenylpropanoid metabolic process” and “cellular amino acid derivative metabolic process” although less than 10% of the 138 genes belonged to these categories (Figure 6b,d). Overall, we found that *TT2* and *MYB5* redundantly control stress response genes while only *TT2* regulates late flavonoid biosynthesis genes. 

We then looked for motif enrichment in the promoters of DEGs. De novo motif discovery was performed using 500 bp upstream of all genes upregulated by TT2 (Figure 6e). Notably, SIIE motif was found to be enriched together with motifs similar to the E-box (Figure 6e). Similar results were obtained from MYB5 upregulated genes. We confirmed this result by looking for enrichment of the motifs in the promoters of genes upregulated in *TT2* OE or *MYB5* OE and found the SIIE was enriched in both cases (*p* = 1.32 × 10^−17^ and *p* = 6.91 × 10^−8^, respectively). Promoters of *TT2* OE downregulated genes were also found to be enriched in SIIE (*p* = 1.09 × 10^−2^). These results further confirm the importance of the SIIE and E-box motif for *TT2*/*MYB5* regulation of transcription. 

### 3.7. TT2 Differentially Regulates Multiple Stress Response and Flavonoid Metabolism Genes 

As *TT2* is a more potent transcriptional regulator than *MYB5*, we used the DEG list of *TT2* to perform gene network analyses. We first performed a GO term enrichment analysis (agriGO; http://bioinfo.cau.edu.cn/agriGO, accessed on August 2017). Enriched GO terms in up- and downregulated genes are presented in Supplementary Tables S5 and S6. Similarly, to the GO term enrichment analysis performed on the *TT2/MYB5* overlap, we found *TT2* OE upregulated genes were strongly enriched in GO categories related to stress and secondary metabolic process. In particular, *HSFA3*, *HSFA7A*, and *MBF1C* (MULTIPROTEIN BINDING 1 C), three major regulators of multiple stress responses, were significantly upregulated (Table 1). Downregulated genes were mostly enriched in phenylpropanoid metabolic process but also showed enrichment in stress response-related terms (Table 2). 

**Figure 7:**
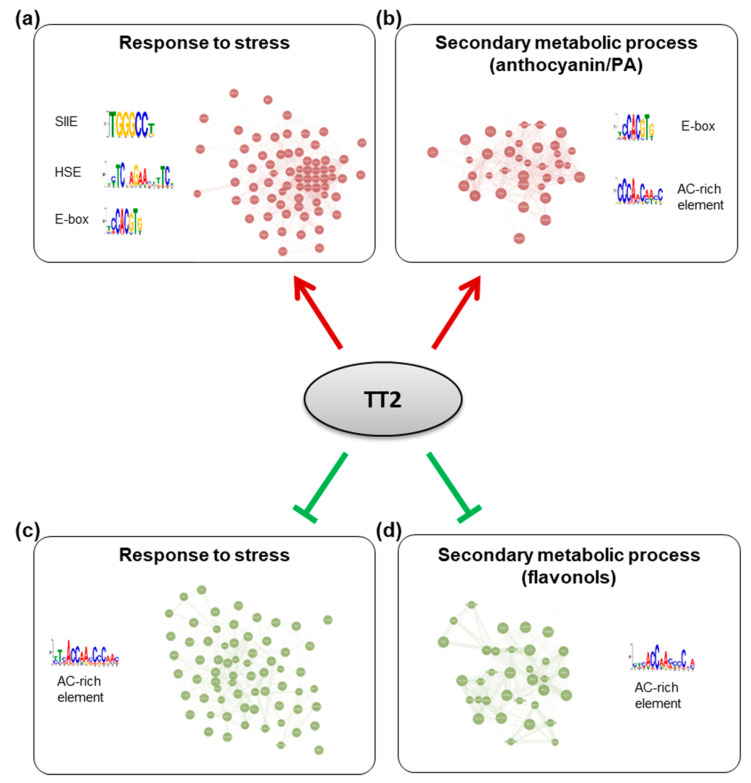
Gene regulatory networks (GRNs) regulated by TRANSPARENT TESTA 2 (TT2) overexpressors (OE). GRNs were constructed from differentially expressed genes in *TT2* OEs. (**a**) Upregulated genes belonging to the “response to stress” gene ontology (GO) category belong to a tightly coregulated set of genes including *HEAT SHOCK FACTOR A2*, *A3*, *A7A*, *MULTIPROTEIN BRIDGING FACTOR 1C*, and many chaperones. Promoters of these genes were found to be enriched in SII element, heat shock element and E-box motifs. (**b**) Upregulated genes belonging to the “secondary metabolic process” GO category formed another network including multiple flavonoids biosynthesis genes (*BANYULS*, *LEUCOANTHOCYANIDIN DIOXYGENASE*, *TRANSPARENT TESTA 5* and *6*, etc…). Promoters of these genes were enriched in E-box and a motif similar to the AC-rich element. A comparable AC-rich element motif was enriched in downregulated GRNs (**c**,**d**).

To further characterize the TT2 DEGs, we used Genemania (https://genemania.org, accessed on August 2017) to build gene regulatory networks (GRN) from these categories. Two major networks of highly co-regulated genes could be defined from the upregulated genes, GRNs “response to stress” and “secondary metabolic process” (Figure 7, Supplementary Table S7). The stress response GRN contained genes involved in response to biotic stress (defense response to bacterium) and multiple abiotic stresses (oxidative, salt, heat, high light, and cold stress), together with genes involved in multiple hormones response (Table 1, Supplementary Tables S7 and S8). Seventeen genes of the *HSFA2* common stress cluster were found in the stress response GRN (Supplementary Table S7). Secondary metabolic process GRN was found mostly involved in the production of PA (Table 1, Supplementary Table S7 and S9). Two GRNs of highly co-regulated genes were found downregulated in *TT2* OE (Figure 7, Supplementary Table S7). The downregulated GRN “response to stress” contained genes related to fungus response together with genes involved in cold, osmotic, and wounding stress response (Supplementary Table S7 and S10). The downregulated GRN “secondary metabolic process” contained genes involved in flavonols biosynthesis, lignin biosynthesis, and early steps of the general phenylpropanoid metabolic pathway (Supplementary Table S7 and S11). 

To screen for shared features between the promoters of the different GRNs, we used the MEME suite V4.12.0 tool to perform de novo motif enrichment analysis. Promoters of upregulated genes from the stress response GRN were found enriched in motifs similar to the SIIE, the E-box, and in HSE (Figure 7). Interestingly, promoters of genes belonging to the secondary metabolism GRN were enriched in E-box motif and in AC-rich element although with high E-values (Figure 7). Promoters of downregulated GRNs were only enriched in motifs corresponding to AC-rich element. 

Overall, *TT2* performs a complex modulation of both stress responses and anthocyanin/PA biosynthesis genes and this modulation seems to involve different cis-elements. 

## 4. Discussion

### 4.1. Role of the TT2/MYB5 in the Plant Heat Stress Response 

In this report, we provide strong evidence for the involvement of TT2 and MYB5 in the heat stress response. We show in transient and stable transformation experiments that TT2 mediates *HSFA2* regulation directly or indirectly via SIIE motifs (Figure 2 and Figure 4). We also show that *TT2* and *MYB5* overexpressing plantlets are heat stress resistant, whereas *tt2*, *myb5*, and *tt2/myb5* loss of function mutants are impaired in heat stress resistance (Figure 3 and Figure 4, Supplementary Figure S2). These results extend previous reports of *tt2* mutant seeds, being more sensitive to salt, sucrose, and ABA that were attributed to an increased permeability of the seed coat to stressors [29]. However, as *myb5*-1 does not exhibit the yellow seed phenotype but show the same sensitivity to heat stress, the hypothesis of the increased permeability of the seed coat to stressors is unlikely (Figure 4). In addition, the significant modulation of stress-related genes in OE plants further establishes a role of *TT2* and *MYB5* as regulators of stress response (Figure 6 and Figure 7). 

TT2 and MYB5 were previously thought to be seed coat-specific factors whereas PAP1 was found to be present in vegetative organs. Here, we show that *TT2* is expressed at low levels in vegetative parts of the plant and that the expression of *TT2*, *BAN*, and *TT8* is modulated by heat stress (Figure 5). Consistent with previous reports [12], *BAN*, *DFR*, *LDOX*, *TT4*, and *TT8* were also induced constitutively in *TT2* OE lines (Figure 4, Table 1 and Supplementary Table S4). These results confirm that the full MBW complex is present and active in vegetative organs. It would be very interesting to localize the tissues where *TT2* and *TT8* are induced during stress to better understand their precise functions. Previously, the PAP1-4-containing MBW complex was thought to be the only MBW complex present in vegetative organs. In our study, *PAP1* was found to be downregulated by heat stress in wild type plants, while *LDOX* and *DFR* were not induced, suggesting that a PAP1-containing MBW is not involved in the heat response (Figure 5). Overall, our results indicate that *TT2* and *MYB5* are genuinely involved in heat stress resistance in vegetative organs, warranting further investigations into the role and mechanisms of MBW complex formation and functioning in different stress conditions.

### 4.2. TT2/MYB5 in Stress Resistance Is Independent of Anthocyanin/PA Metabolism

The MBW complex was demonstrated to regulate anthocyanin and PA accumulation. Flavonoids have been described as important components of multiple biotic and abiotic stress responses [31]. However, we do not believe that these molecules play a major role in the resistance phenotypes associated with *TT2/MYB5* OEs. *TT2* OE alone is not sufficient to trigger ectopic anthocyanin/PA accumulation [32], but is sufficient to increase basal heat stress resistance (Figure 3). In addition, *myb5-1* mutants are not impacted in flavonoid metabolism and do not exhibit the yellow seed phenotype. Still, they show the same defect in thermotolerance as *tt2* mutants. 

Other groups reported 35S:*TT2* plants were inducing strong *BAN* expression without exhibiting PA and PA precursor accumulation in vegetative organs [12]. In our study, we found the PA biosynthesis genes *DFR*, *LDOX*, *BAN* to be specifically upregulated in *TT2* OE plants, while flavonol (*FLS1*) and anthocyanin (*GSTs*) biosynthesis genes were downregulated (Table 1 and Table 2). At the same time, several enzymes of the very early steps of the general phenylpropanoid metabolic pathway were downregulated (*4CL1*, *PAL1* and *PAL4*; Table 2 and Supplementary Table S4). These results most likely explain the absence of PA accumulation in *TT2* OE plants and show that PA accumulation requires other seed-specific factors. 

Gene expression analysis further supports the view of *TT2* functioning in stress resistance and in flavonoid biosynthesis through independent mechanisms. RNA-seq analysis performed in this study suggests that *MYB5* and *TT2* redundantly regulate stress response genes but not flavonoid biosynthesis genes, which were found to be differentially regulated in *TT2* OE plants only. This suggests stress resistance is not the consequence of anthocyanin/PA accumulation. Previous studies of developing *tt2-5* seeds showed that 33.6% and 32.1% of up- and down-regulated genes, respectively, were involved in stress/defense response, whereas only 0.2% and 5.4% of up- and down-regulated genes were involved in flavonoid metabolism [33]. Our findings that *TT2* OE upregulates the expression of many stress-related genes rule out the possibility that this modulation is due to seed coat defects or an overall lack of anthocyanins/PA (Figure 6). Motif enrichment analyses also suggest the regulation of stress response and secondary metabolic process is performed directly, or indirectly, by different protein complexes. SIIE motifs were found to be enriched in the promoters of stress response genes but not in the promoters of secondary metabolic process-related genes (Figure 7). Consequently, we attribute enhanced stress resistance to the modulation of SIIE-regulated genes in general. 

### 4.3. TT2 Dual Modes of Transcriptional Regulation 

TT2 and MYB5 binding sites have been previously identified as the MYB core and AC-rich elements [34]. Contrary to what we observe for *HSFA2* promoter regulation, the *BAN* promoter, which does not contain SIIEs, could not be activated by TT2 or MYB5 alone but required the full MBW complex for proper activation [22]. This indicates that the regulation of *HSFA2* and *BAN* promoters by TT2/MYB5-TT8-TTG1 involves different mechanisms. Examining the *HSFA2* promoter, we found several putative binding sites for TT8 (E-boxes), and MYBs (AC-rich elements, Figure 6), which may explain the residual activation of the *HSFA2* promoter mutated in the SIIEs (Figure 4). Indeed, a combination of TT2-TT8-TTG1 but not MYB5-TT8-TTG1 retained a strong ability to activate the mutated *HSFA2* promoter. This tends to indicate that TT2 and MYB5 alone regulate transcription through SIIEs while TT2-TT8-TTG1 but not MYB5-TT8-TTG1 can activate transcription through other means. Accordingly, GRNs upregulated by *TT2* OE were linked with different cis-regulatory elements (Figure 7). Stress response GRN was linked with HSE, SIIE, and E-box motifs, whereas secondary metabolic process GRN was linked to the E-box and AC-rich element. 

**Figure 8:**
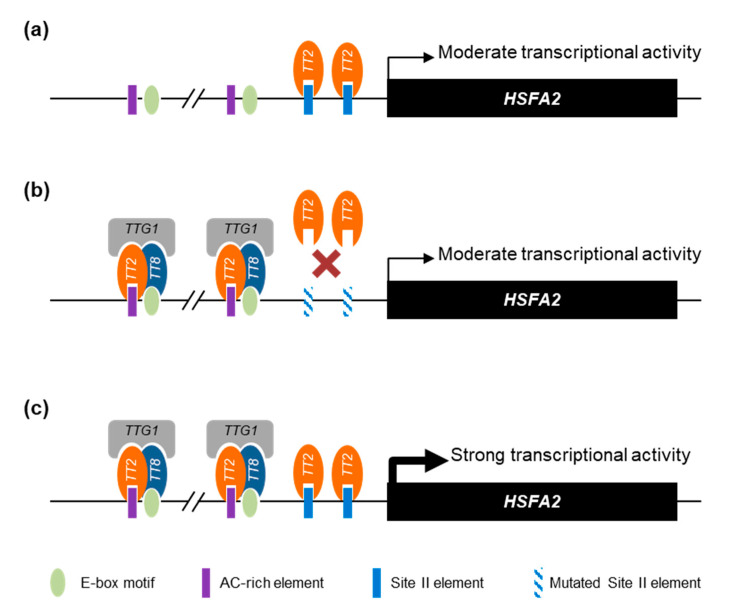
Proposed model of *HEAT SHOCK FACTOR A2* (*HSFA2*) promoter regulation by TRANSPARENT TESTA 2 (TT2) and MYB5. (**a**) When expressed alone, TT2 and MYB5 are able to drive a moderate transcriptional activity mostly dependent on the SII element (SIIE). (**b**) Together with the other members of the MYB–BHLH–WDR (MBW) complex, TRANSPARENT TESTA 8 (TT8) TRANSPARENT TESTA GLABRA 1 (TTG1), TT2 acquires new DNA binding specificities. When the SIIE is mutated, the MBW complex is still able to induce a moderate transcriptional activity through the E-box and AC-rich element motifs. (**c**) The concerted action of TT2-TT8-TTG1 and TT2 or MYB5 monomers trigger a strong activation of the *HSFA2* promoter. For simplicity, we represent the different proteins as regulating transcription through direct DNA binding.

Overall, we propose that the *HSFA2* promoter is regulated by a combination of TT2/MYB5 through SIIEs and the MBW complex through AC-rich and E-box motifs. Interestingly, the *HSFA2* promoter has two tandem repeats of AC-rich elements and E-box motifs separated by exactly 21 nucleotides. Our proposed model of TT2 and MYB5 involvement in *HSFA2* promoter regulation is presented in Figure 8. 

## Figures and Tables

**Table 1:** Selection of *TRANSPARENT TESTA 2* overexpressor upregulated genes from the two major gene ontology categories, response to stress and secondary metabolic process.

Identifier	Fold Change	Gene Description
**Response to Stress**		
**AT5G03720**	1.57	HEAT SHOCK TRANSCRIPTION FACTOR A3 (HSFA3)
**AT3G51910**	1.33	HEAT SHOCK TRANSCRIPTION FACTOR A7A (HSFA7A)
**AT3G24500**	1.39	MULTIPROTEIN BRIDGING FACTOR 1C (MBF1C)
**AT3G16770**	1.52	ETHYLENE RESPONSE FACTOR 72 (ERF72)
**AT5G59780**	1.28	MYB DOMAIN PROTEIN 59 (MYB59)
**AT4G31800**	1.46	WRKY DNA-BINDING PROTEIN 18 (WRKY18)
**AT5G52640**	1.29	HEAT SHOCK PROTEIN 90.1 (HSP90.1)
**AT2G19310**	1.27	HSP20-like chaperones superfamily protein
**AT5G51440**	1.57	HSP20-like chaperones superfamily protein
**AT1G63750**	1.55	Disease resistance protein (TIR-NBS-LRR class) family
**AT1G63860**	1.42	Disease resistance protein (TIR-NBS-LRR class) family
**AT5G41740**	1.45	Disease resistance protein (TIR-NBS-LRR class) family
**AT1G75830**	3.04	Plant defensin 1.1 (PDF1.1)
**AT4G22212**	1.41	Encodes a defensin-like (DEFL)
**AT2G43535**	1.39	Encodes a defensin-like (DEFL)
**AT2G21490**	2.24	dehydrin LEA
**AT2G47180**	1.29	Galactinol synthase 1 (GolS1)
**AT4G11650**	1.57	OSMOTIN 34 (OSM34)
**AT5G66400**	1.32	RESPONSIVE TO ABA 18 (RAB18)
**Secondary Metabolic Process**		
**AT1G71030**	1.30	MYB-LIKE 2 (MYBL2)
**AT5G11260**	1.39	ELONGATED HYPOCOTYL 5 (HY5)
**AT5G13930**	2.15	TRANSPARENT TESTA 4 (TT4)
**AT5G42800**	34.65	DIHYDROFLAVONOL 4-REDUCTASE (DFR)
**AT4G22880**	6.34	LEUCOANTHOCYANIDIN DIOXYGENASE (LDOX)
**AT1G61720**	451.56	BANYULS (BAN)

**Table 2:** Selection of *TRANSPARENT TESTA 2* overexpressors downregulated genes from the two major gene ontology categories, response to stress and secondary metabolic process.

Identifier	Fold Change	Gene Description
**Response to Stress**		
**AT5G08790**	−1.48	ARABIDOPSIS NAC DOMAIN CONTAINING PROTEIN 81 (ANAC81)
**AT5G06960**	−1.30	TGACG MOTIF-BINDING FACTOR 5 (TGA5)
**AT2G14610**	−30.09	PATHOGENESIS-RELATED GENE 1 (PR 1)
**AT2G17060**	−1.97	Disease resistance protein (TIR-NBS-LRR class)
**AT5G07390**	−1.64	RESPIRATORY BURST OXIDASE HOMOLOG A (RBOHA)
**AT5G08590**	−1.39	SNF1-RELATED PROTEIN KINASE 2.1 (SNRK2.1)
**AT1G55020**	−2.42	LIPOXYGENASE 1 (LOX1)
**AT2G37040**	−1.44	PHE AMMONIA LYASE 1 (PAL1)
**AT1G45145**	−1.49	THIOREDOXIN H-TYPE 5 (TRX5)
**AT1G05250**	−13.72	PEROXIDASE 2 (PRX2)
**AT5G51890**	−1.30	PEROXIDASE 66 (PRX66)
**AT2G38380**	−1.47	Peroxidase superfamily protein
**AT1G68850**	−1.80	Peroxidase superfamily protein
**AT5G64110**	−1.30	Peroxidase superfamily protein
**AT5G08670**	−1.35	Encodes the mitochondrial ATP synthase beta-subunit
**AT5G08680**	−1.35	Encodes the mitochondrial ATP synthase beta-subunit
**Secondary Metabolic Process**		
**AT5G08640**	−1.72	FLAVONOL SYNTHASE 1 (FLS1)
**AT5G25980**	−2.88	THIOGLUCOSIDE GLUCOHYDROLASE 2 (TGG2)
**AT5G26000**	−1.35	THIOGLUCOSIDE GLUCOHYDROLASE 1 (TGG1)
**AT1G17170**	−1.47	GLUTATHIONE S-TRANSFERASE TAU 24 (GSTU24)
**AT1G17180**	−2.02	GLUTATHIONE S-TRANSFERASE TAU 25 (GSTU25)
**AT1G78340**	−1.54	GLUTATHIONE S-TRANSFERASE TAU 22 (GSTU22)
**AT1G51680**	−1.28	4-COUMARATE:COA LIGASE 1 (4CL1)
**AT2G37040**	−1.44	PHENYLALANINE AMMONIA LYASE 1 (PAL1)
**AT3G10340**	−1.44	PHENYLALANINE AMMONIA-LYASE 4 (PAL4)

## Data Availability

All the data supporting the results of this paper are present in the paper and/or the supplementary materials. The RNA-Seq data analysed in this article have been deposited in NCBI’s Gene Expression Omnibus (GEO) and are accessible through GEO Series accession number GSE171922 (https://www.ncbi.nlm.nih.gov/geo/query/acc.cgi?acc=GSE171922). All the materials and relevant data are available from the corresponding author on request.

## Supplementary Materials

The following are available online at https://www.mdpi.com/article/10.3390/genes12050746/s1, Figure S1: Graphical representation of the promoter of the 43 coregulated genes showing enriched DNA motifs, Figure S2: *TT2* and *MYB5* are involved in plant stress resistance, Table S1. Genes strongly coregulated with HSFA2 during heat, cold, hypoxia, and salt stresses, Table S2. Genes differentially expressed in TT2 OE or MYB5 OE compared to wild-type plants, Table S3. GO terms enriched in TT2OE upregulated genes, Table S4. GO terms enriched in TT2OE downregulated genes. Table S5. GRNs controlled by TT2, Table S6. GO terms enrichment in upregulated GRN “response to stress”, Table S7. GO terms enrichment in upregulated GRN “secondary metabolic process”, Table S8. GO terms enrichment in downregulated GRN “response to stress”, Table S9. GO terms enrichment in downregulated GRN “secondary metabolic process”, Table S10. Primer used for Y2H prey cloning, Table S11. Primer used for expression analysis.
