# Supplementary material for: The Seed Development Factors TT2 and MYB5 Regulate Heat Stress Response in Arabidopsis
Source: Genes (Basel). 2021 May 15;12(5):746. doi: 10.3390/genes12050746 (PMC8156827; doi:10.3390/genes12050746)
Supplement: Supplementary file 1 [file genes-12-00746-s001.zip › Supplementary data.f.pdf]

Supplementary data:

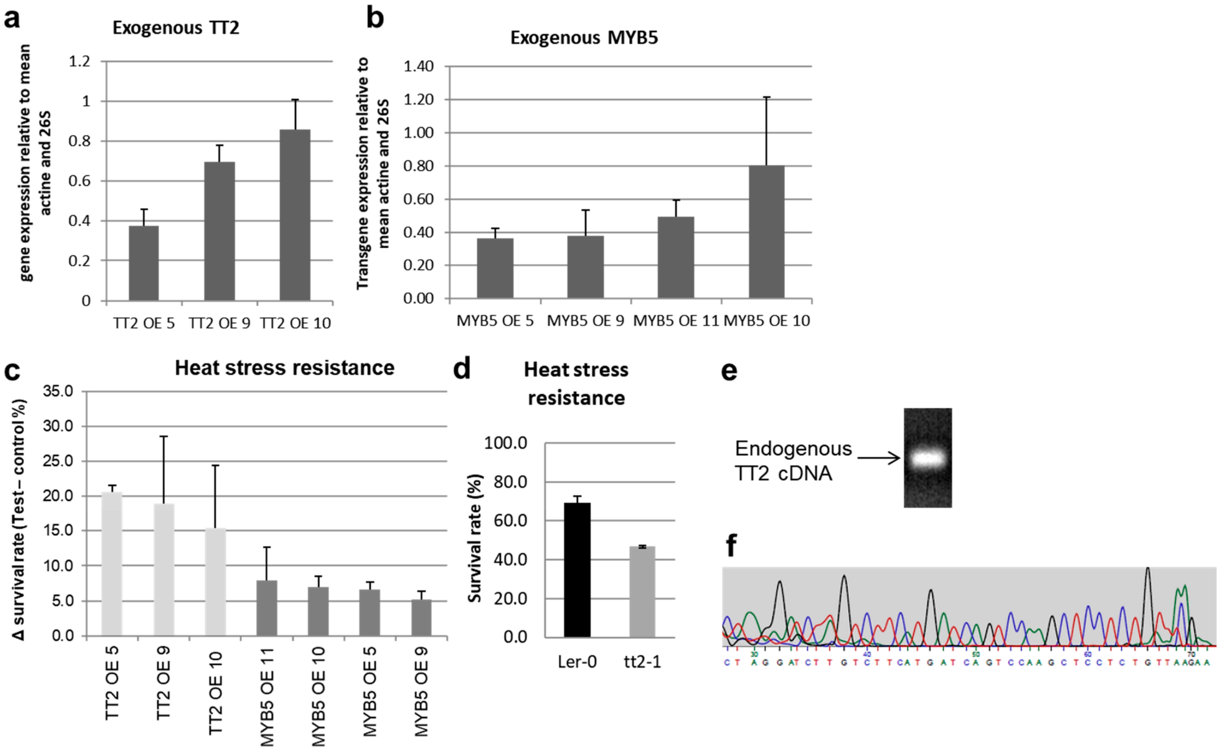

Supplementary Figure S1: TT2 and MYB5 are involved in stress resistance in the whole plant.

a and **b**: Transgene expression analysis in TT2, **a**, and MYB5, **b**, overexpressing lines. Transcripts levels are expressed relative to the mean actine and 26S transcripts levels. **c**: Multiple insertions lines were tested for basal heat stress resistance (80 minutes at 44°C), over three to five independent experiments. Results are expressed as the difference between survival rate in OE lines and in the corresponding Col 0 control. Bars represent SD. The observed resistances are statistically significant with p-values <0,001 (Xhi square test). **d**: Heat stress resistance of tt2-1 mutants compared to Ler 0. Results are statistically significant with p-values <0,001 (Xhi square test). Bars represent SD. **e**: Representative picture showing Q-PCR amplification products of the TT2 mRNA in Col 0 from Fig 5 on a 2% agarose gel. An expected band around 87pb was observed every condition tested. RT-PCR was repeated and the 87bp band was purified and subjected to sanger sequencing. **f**: Portion of the chromatogram indicating the QPCR product dosed in Fig 5 was TT2 mRNA.

Supplementary Table S1: Primer used for prey cloning. Att recombination sequence are presented in lower cases

**Primer name**	**Primer sequence (5'→ 3')**
AT5G35550-f	ggggacaagtttgtacaaaaaagcaggctacATGGGAAAGAGAGCAACTACTAGTG
AT5G35550-r	ggggaccactttgtacaagaaagctgggtgTCAACAAGTGAAGTCTCGGAG
AT3G13540-f	ggggacaagtttgtacaaaaaagcaggctacATGATGTCATGTGGTGGGAA
AT3G13540-r	ggggaccactttgtacaagaaagctgggtgCTAGTCATGTCCTAAGCTAGAAGACG

Supplementary Table S2: Primer used Q-PCR.

Primer name	Primer sequence (5'→ 3')
AT1G61720-F	ACATTTGCTGTGCTTACAACACAAGT
AT1G61720-R	CGAAAGCCTTCATTGATAAGTTTTTGCG
AT2G26150-F	TCCTTCCACGTTACTTCAAGC
AT2G26150-R	CATCCCAGATCCTTGCTGAT
AT3G18780-F	CGTTTCTATGATGCACTTGTGTG
AT3G18780-R	GGGAACAAAAGGAATAAAGAGG
AT4G24820-F	TACCGTTCGGAGTTTCCAAG
AT4G24820-R	AGGTTGCAGGTGTTTTGGAG
AT4G22880-F	AATTGGCTAACAACGCGAGTG
AT4G22880-R	CGTACTCACTCGTTGCTTCTATGTA
AT5G35550-F	AACAGAGGAGCTTGGACTGA
AT5G35550-R	AGGGAGAGTGCTCCATTTGC
AT1G13750-F	CCCTCTTCCTTGACCAATTTACT
AT1G13750-R	TTTAGCCCAGCTCTTACAGGA
AT5G42800-F	GAACGAGGCGCATTACTC
AT5G42800-R	TTGAAGGTACGTTATATTCGGG
AT4G09820-F	GCCATCCACCAAGTCATAATAC
AT4G09820-R	TTTATATGTTGCATCCGGCTAT
35S-terminator-F	AGTGGTTGATAACAGCGGGT
35S-terminator-R	GCAGCGTAATCTGGAACGTC

Supplementary Table S3: List of genes strongly coregulated with HSFA2 during heat, cold, hypoxia and salt stresses. Known HSFA2 targets are presented in red (according to [9]).

Array Element	Locus	Gene description
263164_at	AT1G03070	Glutamate binding /
261838_at	AT1G16030	HSP70B (heat shock protein 70B); ATP binding
260881_at	AT1G21550	Calcium-binding protein, putative
260025_at	AT1G30070	SGS domain-containing protein
262014_at	AT1G35660	Erythroid differentiation factor-like protein
262148_at	AT1G52560	26.5 kDa class I small heat shock protein-like (HSP26.5-P)
260978_at	AT1G53540	17.6 kDa class I small heat shock protein (HSP17.6C-CI) (AA 1-156)
263150_at	AT1G54050	17.4 kDa class III heat shock protein (HSP17.4-CIII)
262911_s_at	AT1G59860; AT1G07400	AT1G59860, 17.6 kDa class I heat shock protein (HSP17.6A-CI); AT1G07400, 17.8 kDa class I heat shock protein (HSP17.8-CI)
256518_at	AT1G66080	Similar to hypothetical protein [Vitis vinifera] (GB:CAN77663.1)
256363_at	AT1G66510	AAR2 protein family
259913_at	AT1G72660	Developmentally regulated GTP-binding protein, putative
260248_at	AT1G74310	ATHSP101 (HEAT SHOCK PROTEIN 101); ATP binding / ATPase
263374_at	AT2G20560	DNAJ heat shock family protein
264402_at	AT2G25140	CLPB-M/CLPB4/HSP98.7 (HEAT SHOCK PROTEIN 98.7); ATP binding / ATPase
266294_at	AT2G29500	17.6 kDa class I small heat shock protein (HSP17.6B-CI)
265675_at	AT2G32120	HSP70T-2; ATP binding
267073_at	AT2G41160	Ubiquitin-associated (UBA)/TS-N domain-containing protein
266590_at	AT2G46240	BAG6 (ARABIDOPSIS THALIANA BCL-2-ASSOCIATED ATHANOGENE 6)
258830_at	AT3G07090	Similar to unknown protein [Arabidopsis thaliana] (TAIR:AT5G25170.1)
258827_at	AT3G07150	Similar to hypothetical protein [Vitis vinifera] (GB:CAN68821.1)
258984_at	AT3G08970	DNAJ heat shock N-terminal domain-containing protein
259037_at	AT3G09350	Armadillo/beta-catenin repeat family protein
258695_at	AT3G09640	APX2 (ASCORBATE PEROXIDASE 2); L-ascorbate peroxidase
256663_at	AT3G12050	Aha1 domain-containing protein
252515_at	AT3G46230	ATHSP17.4 (Arabidopsis thaliana heat shock protein 17.4)
251166_at	AT3G63350	AT-HSFA7B (Arabidopsis thaliana heat shock transcription factor A7B)
254878_at	AT4G11660	AT-HSFB2B (Arabidopsis thaliana heat shock transcription factor B2B); transcription factor
254839_at	AT4G12400	Stress-inducible protein, putative
245272_at	AT4G17250	Similar to unknown protein [Arabidopsis thaliana] (TAIR:AT5G47580.1)
254059_at	AT4G25200	ATHSP23.6-MITO (MITOCHONDRION-LOCALIZED SMALL HEAT SHOCK PROTEIN 23.6)
253884_at	AT4G27670	HSP21 (HEAT SHOCK PROTEIN 21)
250296_at	AT5G12020	HSP17.6II (17.6 KDA CLASS II HEAT SHOCK PROTEIN)
250351_at	AT5G12030	AT-HSP17.6A (Arabidopsis thaliana heat shock protein 17.6A)
250036_at	AT5G18340	U-box domain-containing protein
246944_at	AT5G25450	Ubiquinol-cytochrome C reductase complex 14 kDa protein, putative
246612_at	AT5G35320	Similar to hypothetical protein [Vitis vinifera] (GB:CAN60849.1)
249575_at	AT5G37670	15.7 kDa class I-related small heat shock protein-like (HSP15.7-CI)

Supplementary Table S3 (continued):

Array Element	Locus	Gene description
248774_at	AT5G47830	Similar to hypothetical protein MtrDRAFT_AC150207g26v2 [Medicago truncatula] (GB:ABD32636.1)
248657_at	AT5G48570	Peptidyl-prolyl cis-trans isomerase, putative / FK506-binding protein, putative
248434_at	AT5G51440	23.5 kDa mitochondrial small heat shock protein (HSP23.5-M)
248332_at	AT5G52640	HSP81-1 (HEAT SHOCK PROTEIN 81-1); ATP binding / unfolded protein binding
254414_at	no_match	no_match

Supplementary Table S5: GO term enriched in TT2OE upregulated genes.

GO term	Description	Number of genes	%	FDR-adjusted p-value
GO:0050896	response to stimulus	80	21.3	4.2e-07
GO:0006950	response to stress	53	14.1	2.5e-06
GO:0010876	lipid localization	5	1.3	0.00098
GO:0019748	secondary metabolic process	17	4.5	0.0011
GO:0046283	anthocyanin metabolic process	5	1.3	0.002
GO:0006979	response to oxidative stress	13	3.5	0.0031
GO:0009725	response to hormone stimulus	23	6.1	0.0085
GO:0009812	flavonoid metabolic process	6	1.6	0.0089
GO:0009628	response to abiotic stimulus	30	8	0.0089
GO:0009743	response to carbohydrate stimulus	10	2.7	0.0089
GO:0006952	defense response	19	5.1	0.012
GO:0009408	response to heat	8	2.1	0.012

Supplementary Table S6: GO terms enriched in TT2OE downregulated genes.

GO term	Description	Number of genes	%	FDR-adjusted p-value
GO:0010876	lipid localization	6	1.9	7.1e-05
GO:0019748	secondary metabolic process	17	5.5	0.0002
GO:0009698	phenylpropanoid metabolic process	10	3.2	0.00047
GO:0009607	response to biotic stimulus	16	5.2	0.006
GO:0051704	multi-organism process	17	5.5	0.013
GO:0006950	response to stress	35	11.4	0.013
GO:0051707	response to other organism	14	4.5	0.022
GO:0006519	cellular amino acid and derivative metabolic process	15	4.9	0.022
GO:0050896	response to stimulus	51	16.6	0.025

Supplementary Table S8: GO term enrichment in upregulated GRN "response to stress".

GO term	Description	Number of gene	%	FDR adjusted p-value
GO:0006950	response to stress	65	92,9	1.4e-69
GO:0050896	response to stimulus	65	92,9	2.3e-54
GO:0009628	response to abiotic stimulus	39	55,7	4.9e-34
GO:0009408	response to heat	22	31,4	2.2e-32
GO:0042221	response to chemical stimulus	41	58,6	2.4e-31
GO:0009266	response to temperature stimulus	27	38,6	1.2e-30
GO:0006979	response to oxidative stress	21	30,0	1.8e-24
GO:0010035	response to inorganic substance	20	28,6	2.7e-24
GO:0009642	response to light intensity	14	20,0	4.2e-21
GO:0042542	response to hydrogen peroxide	12	17,1	8.8e-20
GO:0009644	response to high light intensity	12	17,1	1.7e-19
GO:0000302	response to reactive oxygen species	12	17,1	1.2e-17
GO:0006952	defense response	18	25,7	1.1e-13
GO:0010033	response to organic substance	20	28,6	9.4e-12
GO:0009416	response to light stimulus	15	21,4	9.4e-12
GO:0009314	response to radiation	15	21,4	1.3e-11
GO:0009607	response to biotic stimulus	13	18,6	4.6e-09
GO:0009719	response to endogenous stimulus	15	21,4	2.4e-08
GO:0009725	response to hormone stimulus	13	18,6	6.6e-07
GO:0009723	response to ethylene stimulus	7	10,0	2.3e-06
GO:0009605	response to external stimulus	9	12,9	2.3e-06
GO:0051707	response to other organism	10	14,3	3.3e-06
GO:0010038	response to metal ion	7	10,0	6.4e-06
GO:0009617	response to bacterium	7	10,0	7.8e-06
GO:0006970	response to osmotic stress	8	11,4	1.7e-05
GO:0046686	response to cadmium ion	6	8,6	1.9e-05
GO:0051704	multi-organism process	10	14,3	2.7e-05
GO:0009751	response to salicylic acid stimulus	6	8,6	3.4e-05
GO:0009753	response to jasmonic acid stimulus	6	8,6	4.9e-05
GO:0006955	immune response	7	10,0	7.7e-05
GO:0009651	response to salt stress	7	10,0	7.7e-05
GO:0002376	immune system process	7	10,0	7.7e-05
GO:0009743	response to carbohydrate stimulus	6	8,6	7.9e-05
GO:0009737	response to abscisic acid stimulus	7	10,0	8.6e-05

Supplementary Table S8 (continued):

GO term	Description	Number of gene	%	FDR adjusted p-value
GO:0042742	defense response to bacterium	5	7,1	0.00026
GO:0050789	regulation of biological process	19	27,1	0.00034
GO:0009611	response to wounding	5	7,1	0.00039
GO:0009409	response to cold	6	8,6	0.00039
GO:0045087	innate immune response	6	8,6	0.00051
GO:0065007	biological regulation	20	28,6	0.00051
GO:0009415	response to water	5	7,1	0.00091
GO:0050794	regulation of cellular process	17	24,3	0.0011
GO:0006457	protein folding	5	7,1	0.0016
GO:0051716	cellular response to stimulus	7	10,0	0.0088
GO:0007165	signal transduction	8	11,4	0.018
GO:0080090	regulation of primary metabolic process	10	14,3	0.027
GO:0031323	regulation of cellular metabolic process	10	14,3	0.036

Supplementary Table S9: GO term enrichment in upregulated GRN "secondary metabolic process".

GO term	Description	Number of gene	%	FDR adjusted p-value
GO:0019748	secondary metabolic process	32	86,5	7.2e-53
GO:0009404	toxin metabolic process	11	29,7	5.8e-21
GO:0009407	toxin catabolic process	11	29,7	5.8e-21
GO:0044237	cellular metabolic process	34	91,9	3.5e-17
GO:0009698	phenylpropanoid metabolic process	11	29,7	7.5e-16
GO:0009699	phenylpropanoid biosynthetic process	10	27,0	6.5e-15
GO:0008152	metabolic process	34	91,9	1.3e-14
GO:0009987	cellular process	34	91,9	2.3e-13
GO:0006575	cellular amino acid derivative metabolic process	11	29,7	2.3e-13
GO:0009812	flavonoid metabolic process	8	21,6	3,00E-13
GO:0042398	cellular amino acid derivative biosynthetic process	10	27,0	4.4e-13
GO:0019438	aromatic compound biosynthetic process	10	27,0	4.7e-13
GO:0006725	cellular aromatic compound metabolic process	11	29,7	1.8e-12
GO:0009813	flavonoid biosynthetic process	7	18,9	1.1e-11
GO:0044248	cellular catabolic process	12	32,4	4.9e-11
GO:0006519	cellular amino acid and derivative metabolic process	11	29,7	4,00E-10
GO:0006722	triterpenoid metabolic process	5	13,5	4.2e-10
GO:0006721	terpenoid metabolic process	7	18,9	4.8e-10
GO:0046283	anthocyanin metabolic process	5	13,5	1.8e-09
GO:0006720	isoprenoid metabolic process	7	18,9	3,00E-09
GO:0009056	catabolic process	12	32,4	2,00E-08
GO:0042440	pigment metabolic process	6	16,2	3.6e-08
GO:0009411	response to UV	5	13,5	1.7e-07
GO:0044249	cellular biosynthetic process	19	51,4	1.8e-07
GO:0016114	terpenoid biosynthetic process	5	13,5	2.9e-07
GO:0009058	biosynthetic process	19	51,4	3,00E-07
GO:0046148	pigment biosynthetic process	5	13,5	6.2e-07
GO:0008299	isoprenoid biosynthetic process	5	13,5	1.5e-06
GO:0044255	cellular lipid metabolic process	7	18,9	1.2e-05
GO:0044238	primary metabolic process	22	59,5	2.1e-05
GO:0009628	response to abiotic stimulus	9	24,3	5,00E-05
GO:0006629	lipid metabolic process	7	18,9	8,00E-05
GO:0008610	lipid biosynthetic process	5	13,5	0.00034

Supplementary Table S9 (continued):

GO term	Description	Number of gene	%	FDR adjusted p-value
GO:0050896	response to stimulus	13	35,1	0.00035
GO:0009416	response to light stimulus	5	13,5	0.0013
GO:0009314	response to radiation	5	13,5	0.0014
GO:0042221	response to chemical stimulus	8	21,6	0.0034
GO:0006950	response to stress	8	21,6	0.0067

Supplementary Table S10: GO term enrichment in downregulated GRN “response to stress”.

GO term	Description	Number of gene	%	FDR adjusted p-value
GO:0006950	response to stress	42	87,5	3.2e-43
GO:0050896	response to stimulus	42	87,5	1.7e-33
GO:0006952	defense response	16	33,3	4.1e-14
GO:0006979	response to oxidative stress	12	25,0	4,00E-13
GO:0042221	response to chemical stimulus	20	41,7	5.3e-12
GO:0009607	response to biotic stimulus	13	27,1	2.2e-11
GO:0009611	response to wounding	9	18,8	9,00E-11
GO:0009605	response to external stimulus	11	22,9	1.1e-10
GO:0051707	response to other organism	11	22,9	3.1e-09
GO:0051704	multi-organism process	11	22,9	4.1e-08
GO:0009628	response to abiotic stimulus	13	27,1	2.8e-07
GO:0009620	response to fungus	6	12,5	7.3e-07
GO:0009698	phenylpropanoid metabolic process	6	12,5	1.2e-06
GO:0009409	response to cold	7	14,6	2.1e-06
GO:0009266	response to temperature stimulus	7	14,6	2.5e-05
GO:0006575	cellular amino acid derivative metabolic process	6	12,5	2.8e-05
GO:0006631	fatty acid metabolic process	5	10,4	9.1e-05
GO:0006725	cellular aromatic compound metabolic process	6	12,5	9.3e-05
GO:0019748	secondary metabolic process	6	12,5	0.00027
GO:0032787	monocarboxylic acid metabolic process	5	10,4	0.0012
GO:0006970	response to osmotic stress	5	10,4	0.0012
GO:0006519	cellular amino acid and derivative metabolic process	6	12,5	0.0014
GO:0044255	cellular lipid metabolic process	5	10,4	0.007
GO:0010033	response to organic substance	7	14,6	0.0085
GO:0019752	carboxylic acid metabolic process	5	10,4	0.024
GO:0006082	organic acid metabolic process	5	10,4	0.024
GO:0006629	lipid metabolic process	5	10,4	0.024
GO:0043436	oxoacid metabolic process	5	10,4	0.024
GO:0042180	cellular ketone metabolic process	5	10,4	0.026

Supplementary Table S11: GO term enrichment in downregulated GRN “secondary metabolic process”.

GO term	Description	Number of gene	%	FDR adjusted p-value
GO:0019748	secondary metabolic process	26	70,3	4.2e-39
GO:0009698	phenylpropanoid metabolic process	17	45,9	2.2e-28
GO:0009699	phenylpropanoid biosynthetic process	15	40,5	1.7e-25
GO:0006575	cellular amino acid derivative metabolic process	17	45,9	1.5e-24
GO:0006725	cellular aromatic compound metabolic process	17	45,9	6,00E-23
GO:0042398	cellular amino acid derivative biosynthetic process	15	40,5	1.1e-22
GO:0019438	aromatic compound biosynthetic process	15	40,5	1.2e-22
GO:0006519	cellular amino acid and derivative metabolic process	17	45,9	2.5e-19
GO:0009813	flavonoid biosynthetic process	7	18,9	1,00E-11
GO:0009812	flavonoid metabolic process	7	18,9	2.1e-11
GO:0009407	toxin catabolic process	6	16,2	2,00E-10
GO:0009404	toxin metabolic process	6	16,2	2,00E-10
GO:0044237	cellular metabolic process	26	70,3	5.3e-09
GO:0009611	response to wounding	7	18,9	7.2e-09
GO:0008152	metabolic process	26	70,3	4,00E-07
GO:0009605	response to external stimulus	7	18,9	1.2e-06
GO:0044248	cellular catabolic process	8	21,6	2.8e-06
GO:0009987	cellular process	26	70,3	2.8e-06
GO:0044249	cellular biosynthetic process	16	43,2	2.3e-05
GO:0009058	biosynthetic process	16	43,2	3.7e-05
GO:0050896	response to stimulus	14	37,8	5.7e-05
GO:0009056	catabolic process	8	21,6	0.00014
GO:0044238	primary metabolic process	20	54,1	0.00021
GO:0009416	response to light stimulus	5	13,5	0.0012
GO:0006950	response to stress	9	24,3	0.0012
GO:0009314	response to radiation	5	13,5	0.0013
GO:0009628	response to abiotic stimulus	7	18,9	0.0018
